# Episodic Ataxias: Primary and Secondary Etiologies, Treatment, and Classification Approaches

**DOI:** 10.5334/tohm.747

**Published:** 2023-03-28

**Authors:** Anhar Hassan

**Affiliations:** 1 Beacon Hospital, Sandyford, Dublin 18, Ireland; 2 Trinity College Dublin, Dublin 2, Ireland

**Keywords:** ataxia, cerebellum, episodic, paroxysmal, genetic, primary, secondary, treatment

## Abstract

**Background:**

Episodic ataxia (EA), characterized by recurrent attacks of cerebellar dysfunction, is the manifestation of a group of rare autosomal dominant inherited disorders. EA1 and EA2 are most frequently encountered, caused by mutations in *KCNA1* and *CACNA1A*. EA3–8 are reported in rare families. Advances in genetic testing have broadened the *KCNA1* and *CACNA1A* phenotypes, and detected EA as an unusual presentation of several other genetic disorders. Additionally, there are various secondary causes of EA and mimicking disorders. Together, these can pose diagnostic challenges for neurologists.

**Methods:**

A systematic literature review was performed in October 2022 for ‘episodic ataxia’ and ‘paroxysmal ataxia’, restricted to publications in the last 10 years to focus on recent clinical advances. Clinical, genetic, and treatment characteristics were summarized.

**Results:**

EA1 and EA2 phenotypes have further broadened. In particular, EA2 may be accompanied by other paroxysmal disorders of childhood with chronic neuropsychiatric features. New treatments for EA2 include dalfampridine and fampridine, in addition to 4-aminopyridine and acetazolamide. There are recent proposals for EA9–10. EA may also be caused by gene mutations associated with chronic ataxias (*SCA-14, SCA-27, SCA-42, AOA2, CAPOS*), epilepsy syndromes (*KCNA2, SCN2A, PRRT2*), GLUT-1, mitochondrial disorders (*PDHA1, PDHX, ACO2*), metabolic disorders (Maple syrup urine disease, Hartnup disease, type I citrullinemia, thiamine and biotin metabolism defects), and others. Secondary causes of EA are more commonly encountered than primary EA (vascular, inflammatory, toxic-metabolic). EA can be misdiagnosed as migraine, peripheral vestibular disorders, anxiety, and functional symptoms. Primary and secondary EA are frequently treatable which should prompt a search for the cause.

**Discussion:**

EA may be overlooked or misdiagnosed for a variety of reasons, including phenotype-genotype variability and clinical overlap between primary and secondary causes. EA is highly treatable, so it is important to consider in the differential diagnosis of paroxysmal disorders. Classical EA1 and EA2 phenotypes prompt single gene test and treatment pathways. For atypical phenotypes, next generation genetic testing can aid diagnosis and guide treatment. Updated classification systems for EA are discussed which may assist diagnosis and management.

## Introduction

The term ‘episodic ataxia’ originally refers to a small group of rare autosomal dominant inherited disorders [1]. These are characterized by discrete attacks of cerebellar dysfunction (ataxia) of variable duration and frequency, often accompanied by other ictal and interictal symptoms. The incidence is likely less than 1 per 100,000, but may be underestimated due to restricted genetic testing and unidentified genes [2]. The group comprises eight subtypes (EA 1–8). EA1 and EA2 are the most common subtypes, caused by mutations in *KCNA1* and *CACNA1A* respectively. They are classical neurological channelopathy disorders. They have well-defined phenotypes and are reported in multiple families of different ethnicity. In contrast, EA 3–8 are reported in rare families. Genes for EA5 (*CACNB4*), EA6 (*SLC1A3*) and EA8 (*UBR4*) have been identified, while causative genes are inconclusive for EA3, EA4 and EA7 (either mapped to chromosomal location or unknown) [3].

With increased availability of genetic testing, the phenotypic spectrum of EA1 and EA2 has broadened. Moreover, an increasing number of reports have surfaced of EA as a rare manifestation of other genetic disorders (e.g. epilepsies, paroxysmal dyskinesias, metabolic disorders) [4]. There are also secondary causes of EA that may be encountered, most commonly vascular, multiple sclerosis, or inflammatory disorders. These may be suggested by onset after adolescence, negative family history, greater attack variability, and accompanied by abnormal laboratory and imaging findings [4]. However some clinical features can overlap with primary EAs. There are also a variety of EA mimickers much more commonly encountered, such as migraine or vestibular disorders. Hence there are increasing diagnostic challenges for physicians encountering patients with EA.

This review will focus on providing an update for neurologists and movement disorders specialists regarding clinical and genetic classifications of EA, and a diagnostic and management approach.

## Methods

**Figure 1:**
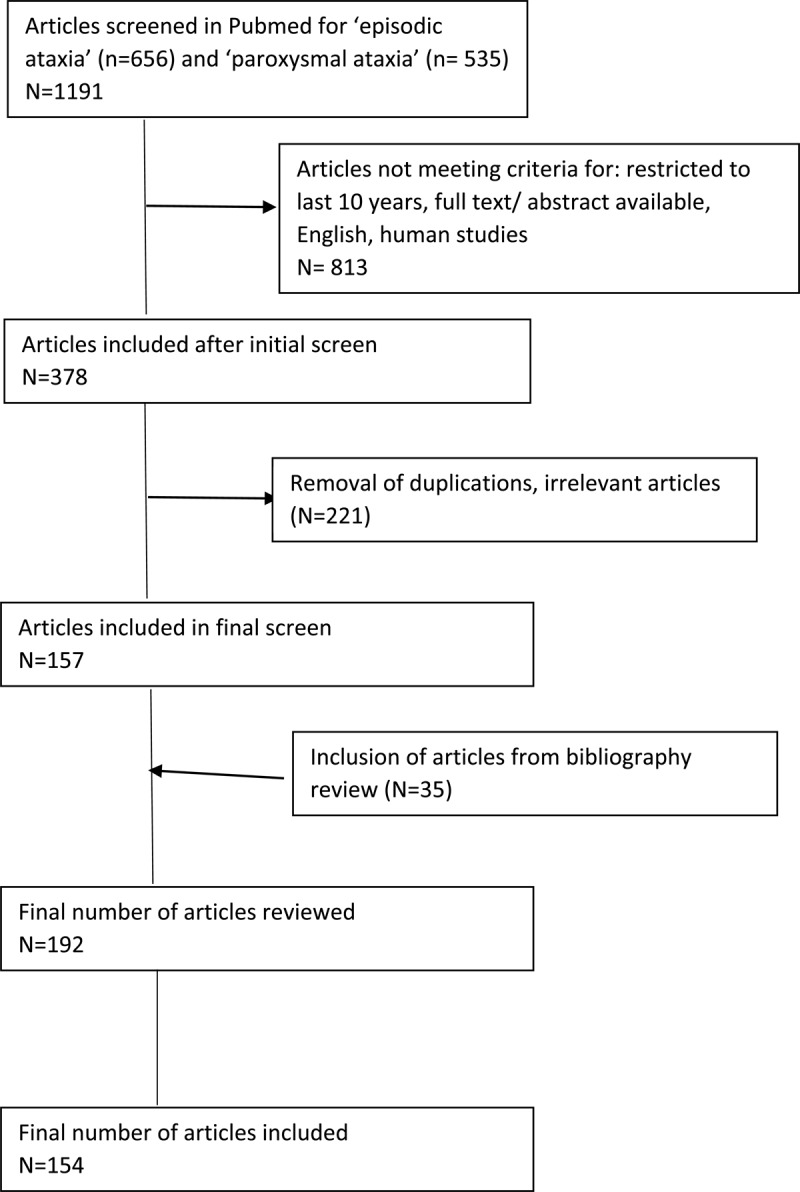
**Flow diagram of literature search.** Summary of steps involved in the literature search leading to final number of articles included.

A systematic literature search of PubMed was performed in October 2022 using the search term ‘episodic ataxia’ (656 articles) and ‘paroxysmal ataxia’ (535 articles). (Figure 1). Restricting the search to manuscripts published within the last 10 years, English language, human subjects, and removal of duplications, yielded 330 articles. After screening titles and abstracts, non-relevant articles were excluded. Of these, 157 reviews, case reports, case series, and literature reviews including systematic reviews were evaluated. Additional articles identified from bibliographic review of screened articles (35 additional articles) resulted in a total of 192 articles reviewed. Of these, 154 were referenced in this review. The author undertook a descriptive analysis, where episodic ataxia was discussed according to the subtype, clinical description, genetics, and treatment modalities.

## Results

### Primary EA

These still formally comprise 8 subtypes, with recent proposals for EA9 and EA10. (Table 1) Amongst EA1–8, there are 5 known genes, and at least 8 loci. All identified genes, except *UBR4* (EA8), encode ion channel proteins, and are important in excitatory neurotransmission [2]. Both EA1 and EA2 are well-established classical channelopathies, with numerous cases/families reported, a known gene comprising numerous mutations, and a narrow classical phenotype. Both EA1 and EA2 have broadened their phenotypes considerably in recent years. However, no fixed genotype-phenotype relationship is identified, and there can be marked clinical variability between family members with the same mutation. Amongst EA3–8, additional cases of EA6 (*SLC1A3*) and EA8 (*UBR4*) were recently found. However, EA3, EA4, EA5 and EA7 remain elusive with no additional cases identified in the past 15–20 years since these were first reported, despite the increased availability of genetic testing. EA 3–8 generally resemble EA1 or EA2 with a few clinical differences, including variability with age of onset (infancy to late adulthood), attack duration (seconds to days), and associated ictal and interictal symptoms.

**Table 1:** Genetic causes of episodic ataxia.


	GENE	PROTEIN	INHERITANCE	OMIM	EA PHENOTYPE	ONSET AGE	ATTACK DURATION	MYOKYMIA	NYSTAGMUS	TINNITUS	EPILEPSY	CHRONIC ATAXIA	OTHER FEATURES	ACETAZOLAMIDE RESPONSE

**Primary EAs**														

EA1	KCNA1	Kv1.1	AD	160120 176260	Brief attacks, interictal myokymia	Child (<age 20)	Secs-Mins	Yes	No	+/–	+/–	+/–	+/–cramps, stiffness, deafness	Variable

EA2	CACNA1A	Cav2.1	AD	108500 601011	Long attacks, interictal nystagmus	Child, adult	Mins-hours (days)	No	Yes	No	+/–	+/–	Migraine (50%)	Yes

EA3	Locus 1q42	Unknown	AD	606554	Like EA1 plus tinnitus	Child, adult	Mins	Yes	+/–	Yes	+/–	No		Yes

EA4	Linkage excluded EA1 & 2	Unknown	AD	606552	Like EA2, no nystagmus	Adult	Hours	No	No	No	+/–	No		

EA5	CACNB4	Cav2.1	AD	613855 601949	Like EA2	Adult	Hours	No	Yes	No	Yes	No		Yes

EA6	SLC1A3	EAAT1	AD	612656 600111	Like EA2	Child, adult	Hours	No	Yes	No	+/–	+/–	+/–migraine, alternating hemiplegia	Yes

EA7	Maps 19q13	Unknown	AD	611907	Like EA2, no nystagmus		Hours	No	No	No	No	No		

EA8	UBR4	Unknown	AD	616055 609890	Overlap with EA1 and EA2	Age 2	Mins-hours	+/–	+/–	No	No	No	Intention tremor	No

EA9?	FGF14	Fibroblast growth factor 14	AD	601515 609307	Like EA2 with tremor, may be brief	Child, adult	Secs-days	No	Yes	No	No	Yes	Vertigo, tremor	Yes

EA9?	SCN2A	Nav1.2	AD	182390 607743		Infant, child	Mins-hours	No	No	No	Yes	No	Neonatal seizures	Variable

EA10?	CACNA1G	VGCC	AD	604065		Adult	Up to months	No	No	No	No	Yes	Facial numbness, movement-induced vertigo, bilateral vestibulopathy	No


#### EA1

##### Clinical

The classical description, first defined in 1975, is brief attacks of ataxia and vertigo [5]. Constant myokymia of the face or limb muscles, detected clinically or via electromyography, affects almost all patients [6]. Onset is typically in childhood, on average at age 7.8 years [6,7]. Attack triggers are numerous; most commonly physiological stressors (exercise, emotional stress, environmental heat, fever, menstruation), caffeine, or alcohol. Sudden movement (kinesogenic trigger), startle, and spontaneous onset are also reported [6,8]. Attacks typically last seconds to minutes, but can last hours or days [5,6,9]. The attack frequency can range from multiple daily attacks to monthly attacks [6,8]. During the attack, gait impairment may range from mild dysfunction to complete inability to walk [6].

##### Genetics

EA1 is mostly familial, although de novo mutations occur [10]. The *KCNA1* gene, discovered in 1994, encodes the fast voltage-gated potassium channel Kv1.1, and mutations result in a potassium channelopathy [3,11]. Kv1.1 is a critical regulator of neuronal excitability in the central and peripheral nervous system, reflecting the neurologic manifestations of EA1. Each Kv1.1 channel is composed of four α-subunits forming a functional transmembrane pore [3]. Each α-subunit has six transmembrane spanning segments (helices S1–S6) and intracellular N- and C- terminal domains. Helices S1–S4 form the voltage-sensing domain, with S4–S5 helical linker to the channel pore. The S5–S6 segments forms the pore region which allows ion flux. RNA editing of *KCNA1* transcripts is important to control protein function. The channels have a low threshold for activation, and a hyperpolarizing effect on membrane potential which limits neuronal excitability. Dysfunction of the channel leads to excessive excitability and increased duration of action potentials, which may cause excessive neurotransmitter release. Kv1.1. is abundant in the brain, mainly in cerebellum, hippocampus, neocortex and peripheral nerves [10]. In EA1, Kv1.1 dysfunction is thought to cause hyperexcitability of cerebellar interneurons, resulting in excessive inhibition of Purkinje cells, which then reduces cerebellar inhibitory output, with subsequent cerebellar deficits [3,5]. To date, 63 *KCNA1* pathogenic mutations are reported on OMIM and most are missense mutations. Functional studies have correlated mutations with Kv1.1 loss-of-function by various mechanisms [3]. Some mutations have a dominant negative effect, meaning that the mutated α-subunit adversely affects the other subunits in the tetrameric structure of the K channel. Other mutations affect Kv1.1 expression or function in other ways [3].

##### Expanded description

Diplopia, dysarthria, nausea, or headache may accompany attacks. Neuromyotonia of variable severity is common, suggested by muscular stiffening, painful contractures, muscle cramps, twitching, or muscle hypertrophy [2]. Other neuromuscular features may include cataplexy, dystonia, distal weakness, and malignant hyperthermia [5,6,9,11,12,13]. Sweating, hot flushes, palpitations, paroxysmal dyspnea, or sensory symptoms are rare features [6,9,14]. Most patients have normal cerebellar function between attacks and normal MRI brain imaging. However longer disease duration is correlated with permanent cerebellar signs and cerebellar atrophy [6]. Epilepsy is more common in EA1 patients than the general population implicating *KCNA1* as a cause of epilepsy [15,16]. There may be comorbid cognitive disability or deafness [8]. Quality of life is impaired, with mental health the worst affected domain [6].

No single phenotype-genotype correlation reported. The same *KCNA1* mutation can show marked clinical variability within the same family or even in twins. This suggests other genetic modifiers or environmental factors influence clinical severity [10,17]. There may be other genes responsible for EA1, as phenocopies (i.e. *KCNA1*-negative cases of EA1) have been identified. The KCNA-negative phenocopies have male predominance and longer attacks versus *KCNA1*-positive cases [6]. Over half of *KCNA1* variants result in EA1, either with or without epilepsy. Other *KCNA1* variants occur without EA, and instead present with epilepsy, epileptic encephalopathy, hypomagnesemia, muscle cramps, myokymia, cataplexy, dystonia or paroxysmal kinesogenic dyskinesia [3,10,18]. In an analysis of 47 pathogenic *KCNA1* mutations, EA-1 associated variants occur along the whole length of the protein, whereas epilepsy-related variants tend to cluster in the S1/S2 transmembrane domains and pore region of Kv1.1 [3]. Research into small molecules that selectively open Kv1.1 channels may permit a future treatment strategy for treating *KCNA1* [19]. In a rat model of focal neocortical epilepsy gene, Kv1.1 overexpression was effective in controlling seizures, although this has not yet been studied for EA1 [20].

##### Management

The diagnosis is based on clinical findings, electrophysiology studies, and genetic confirmation of *KCNA1* mutation. Many patients do not seek treatment because attacks are brief and improve with age [6]. A variety of antiseizure medications can diminish attacks, including carbamazepine, phenytoin, and lamotrigine [1,5,6]. Carbamazepine improved the severity of myokymia, in addition to ataxia, in a patient with a novel *KCNA1* mutation [11]. Acetazolamide and benzodiazepines are helpful in rare cases [10,21]. However medication response is highly variable, and severe drug-resistant phenotypes are encountered [10].

#### EA2

##### Clinical

This is the most common hereditary episodic ataxia. The classical description, first published in 1946, is intermittent spells of ataxia and dysarthria lasting several hours, possibly up to 2–3 days [2,22]. There is interictal nystagmus between attacks, a useful clinical clue. This may be primary position downbeat, gaze-evoked or rebound nystagmus [2,22,23]. The attack triggers include emotional or physiological stress, exercise, alcohol and caffeine [22]. Onset in childhood is most common but it can occur in the sixth decade [2,24]. The dysarthria-ataxia spells may be isolated, or accompanied by other brainstem symptoms (vertigo, diplopia, tinnitus) or other features (migraine, abdominal pain, seizures, dystonia, cognitive impairment) [22,23,25,26]. While initially episodic, some patients develop a progressive cerebellar ataxia syndrome and cerebellar midline atrophy, similar to EA1 [27]. Migraine is reported in up to 50% of cases, and may be hemiplegic migraine [2,22].

##### Genetics

*CACNA1A* was identified as the genetic cause in 1996, and causes a calcium channelopathy [28]. *CACNA1A* encodes the alpha 1A subunit of the P/Q-type voltage-gated calcium channel (Cav2.1). The P/Q channel is expressed throughout the CNS, most densely in cerebellar Purkinje cells and granule layer neurons. It is mainly found on presynaptic terminals and is important for synaptic transmission. *CACNA1A* gene mutations for EA2 have high but incomplete penetrance at 80–90%. Over 100 pathologic mutations are reported to date on OMIM, typically nonsense or frameshift mutations leading to a premature stop in protein transcription. These result in loss of P/Q channel function in the cerebellum. Similar to EA1, the mutation may exert a dominant negative effect [29].

In addition to EA2, *CACNA1A* mutations can result in two other autosomal dominant disorders: familial hemiplegic migraine type 1 (FHM1) and spinocerebellar ataxia type 6 (SCA6). While EA2 is associated with loss-of-function mutations, FHM1 is caused by gain-of-function mutations of the alpha subunit of the Cav 2.1 channel, while SCA6 is caused by a polyglutamine repeat expansion in the alpha subunit [1]. Clinical overlap between EA2, FHM1 and SCA6 is reported. Most FHM1 patients have cerebellar signs and symptoms, while half of EA2 patients have migraine, and SCA6 cases can present with fluctuating ataxia at onset before the progressive cerebellar dysfunction evolves [1]. Consistent with these findings, patients clinically diagnosed with SCA6 may have missense mutations, and EA2 phenotype may have CAG repeat expansions [27,30,31].

##### Expanded description

Episodic weakness may occur during the attack, including generalized weakness, hemiplegic weakness, and ‘myasthenic-like’ weakness associated with variability of neuromuscular transmission on repetitive nerve stimulation [32]. Interictal vestibular impairment has been observed, suggesting degeneration of the vestibulocerebellum or vestibular nuclei [33]. Other eye movement abnormalities have been reported, including slowed abduction during smooth pursuit, slow saccade velocities, and exercise-induced downbeat nystagmus [34,35,36]. Infants and children with CACNA1A mutations may have paroxysmal tonic upgaze, or other paroxysmal eye movement disorders, before developing EA2 attacks in later life [37,38]. Young children can also manifest with benign paroxysmal torticollis of infancy before later developing EA2 [39].

Complicated EA2 syndromes have been reported, where a combination of epilepsy, cognitive impairment, and autism appear in very early childhood, often with cerebellar atrophy, before typical EA2 attacks emerge in later life [27,40,41]. In a cohort of *CACNA1A*-positive infantile-onset disorders, nearly all had congenital cerebellar ataxia or paroxysmal events, frequently with cognitive disorders, followed by epilepsy and cerebellar atrophy after age two [42]. Another study of *CACNA1A* carriers reported absence epilepsy in childhood followed in later life by slowly progressive ataxia without EA [43]. In older patients with *CACNA1A*-positive EA2, neuropsychiatric manifestations could be found dating back to childhood [44]. A small study of children with *CACNA1A* and EA or other paroxysmal events commonly had cognitive impairment, more likely when cerebellar atrophy was present on MRI [45].

Neurophysiological studies may show abnormal signatures for EA2. EEG abnormalities are highly prevalent between attacks, especially in younger patients or with early-onset attacks [46]. Amongst *CACNA1A*-positive adult cases with EA, instrumented gait analysis can detect a specific gait signature of narrow-based gait and lower landing acceleration [47]. In pediatric cases, single fiber EMG can be a useful diagnostic aid to confirm abnormal neuromuscular transmission, where genetic analysis is difficult or novel mutations are present [48].

A single *CACNA1A* mutation can result in widely different phenotypes, even within the same family (e.g. EA, FHM, chronic ataxia, headaches) [26,49,50,51,52,53]. A Korean study suggested possible anticipation in an EA2 family with childhood epilepsy, with splice site mutation and normal repeat number [54]. Increased phenotypic variability and large numbers of variants of uncertain significance (VUS) are becoming challenges in the genetic diagnosis of *CACNA1A*.

##### Management

Diagnosis can be made by a combination of clinical features and confirmed by genetic testing. EA2 is usually distinguished from other EAs by attack duration and interictal nystagmus. However, misdiagnosis as functional ataxia, anxiety, TIA, seizures or migraines has been reported before the correct genetic diagnosis was made [22,55,56,57].

Acetazolamide can reduce or completely abolish attacks, and is a hallmark of the disease. It is a carbonic anhydrase inhibitor but the mode of action is not well understood for EA [58]. About 50–75% patients report improvement with 250 mg to 1000 mg daily. However side effects of nephrolithiasis, paresthesia, and fatigue may limit tolerance [58]. The potassium channel blocker 4-aminopyridine (4-AP) is also effective, reducing the number of attacks and improving quality of life in an RCT [59]. Dalfampridine, a slow release formulation of 4-AP, is also effective for EA2 [60]. In a recent head-to-head trial, both 4-AP 5 mg TID and fampridine (a newer slow release version of 4-AP) 20 mg daily significantly reduced the number of attacks in patients with EA2 and related disorders, in comparison to placebo [61]. Fampridine had fewer side effects than acetazolamide. The combined use of topiramate and 4-AP was effective in a patient with EA2 with migraine that was refractory to acetazolamide [62]. A mouse model of EA2 suggests early treatment with 4-AP may be neuroprotective for secondary progressive ataxia [63]. Levetiracetam is also reported to be beneficial in EA2 [64,65]. Brief naps alleviated attacks in one case of EA2 with migraine, but the attack persisted if they remained awake, suggesting a sleep-neuromodulation effect [66].

#### EA3: (gene unknown)

This was reported in a single large Canadian family in 2001. It resembles EA1, with short attacks with vertigo and tinnitus and interictal myokymia. It may respond to acetazolamide [67]. It is distinguished by EA1 and EA2 by vertigo and tinnitus accompanying the attacks, absent interictal nystagmus, and shorter attacks. Linkage studies excluded *KCNA1* and *CACNA1A* as a cause, and mapped the gene 1q42 with a high LOD score, but only after adapting linkage parameters [68]. Some have questioned the reliability of this finding.

#### EA4: (gene unknown)

This is also termed ‘periodic vestibulocerebellar ataxia’ and ‘North Carolina autosomal dominant ataxia’. It is reported in 2 families from North Carolina, suggesting a common single founder. It is characterized by ataxia, vertigo, episodic impaired smooth pursuit, gaze-evoked nystagmus, and diplopia [69]. The onset is between 30–60 years, and symptoms worsen over time. It resembles EA2 but without interictal nystagmus, and it does not respond to acetazolamide. Gabapentin may relieve vertigo symptoms in EA4 [70]. Linkage studies in 1996 excluded autosomal dominant ataxias with known chromosomal localization at that time, including KCNA1, CACNA1A, SCAs 1–5 and DRPLA [71]. Autopsy findings in a 91-year old EA4 patient showed polyglutamine repeats in Purkinje and granule cells, without intranuclear inclusions, similar to SCA6 brains [72]. This was of interest as SCA6 can present as a fluctuating ataxia.

#### EA5: (*CACNB4*)

This was reported in a single French-Canadian family 20 years ago, with a mutation in *CACNB4* gene, coding for the beta4 auxiliary subunit of voltage-gated calcium channels (Cav2.1) [73]. It is late onset and responds to acetazolamide. There were attacks of vertigo and ataxia lasting for several hours, although 1 family member had a single attack lasting for weeks. Interictal examination revealed spontaneous downbeat and gaze-evoked nystagmus, mild dysarthria and truncal ataxia. However no additional cases have subsequently been identified, despite frequent screening of *CACNB4* mutation in EA patients [74,75]. Meanwhile, the same mutation was reported in a family with epilepsy [20]. Other CACNB4 mutations have been associated with epilepsies [76]. Some researchers have questioned whether there is sufficient data to support EA5 [7].

#### EA6: (*SLC1A3*)

EA6 has been reported in several Caucasian and Korean families to date, and associated with *SLC1A3* mutations in all cases [77,78,79,80,81,82]. The phenotype resembles EA2 with long duration attacks, interictal nystagmus, similar triggers, and acetazolamide response [79]. Migraine, alternating hemiplegia, progressive ataxia and epilepsy may co-occur. Childhood and adult onset is reported, and it appears to have reduced penetrance [74]. The *SLC1A3* gene codes for EAAT1 (excitatory amino acid transporter), a glial Na+–dependent glutamate transporter and ion channel [1,81]. Functional studies suggest *SLC1A3* mutations impair EAAT1 by altering transport function in various ways via reduced or enhanced glutamate uptake and/or anion currents [78,83]. There are differing clinical phenotypes, according to the glutamate reuptake capability [7,74,80]. A *SLC1A3* mutation underlying EA6 has also been reported in a family with adult-onset progressive ataxia [81]. SCL1A3 mutations have also been found in migraine, ADHD, autism and Tourette syndrome. In a patient with familial migraine, the mutation impaired K+ binding to the EAAT1 channel and completely disrupted glutamate transport [84].

#### EA7: (unknown gene)

This was reported in 7 members of a 4-generation family in 2007. It is similar to EA2 but without interictal nystagmus. This was mapped to 19q13 with a LOD score slightly above significance cutoff. Sequencing of 2 candidate genes in this region (*KCNC3, SLC17A7*) did not identify a mutation [85].

#### EA8: (*UBR4*)

This was first reported in 2016 in an Irish 3-generation family [86]. This presented by age 2, much earlier than EAs 1–7. There are episodic attacks with impaired balance, dysarthria, and generalized weakness. Attacks can be triggered by physical fatigue or stress. Interictal examination can show intention tremor, eyelid myokymia, and impaired tandem gait. Attacks vary in duration from minutes to hours, and frequency ranges from daily to every few months. Migraine with aura may co-occur. Attacks respond to clonazepam and are not improved with acetazolamide [86]. The gene has been mapped to a large region on 1p36.13–p34.3 with a LOD score near to cutoff. Exome sequencing revealed variants in 2 genes, SPG2 and *UBR4*. *UBR4* had a greater likelihood of pathogenicity than SPG2, as *UBR4* is ubiquitin ligase protein that interacts with calmodulin and may potentially disrupt calcium sensor in neurons as hypothesis for ataxia [86]. A Korean study reported 2 patients with mutations in both *UBR4* and *CACNA1A*, and suggested *UBR4* may act as a genetic modifier with synergic effects on abnormal *CACNA1A* activity [74]. No functional analysis studies have been performed as yet for *UBR4*.

### Episodic ataxias associated with other genetic disorders

There are a growing number of genetic disorders that can present with EA either alone or embedded in a complex syndrome. These include chronic ataxia disorders (*SCA-14, SCA-27, SCA-35, SCA-42, AOA2, CAPOS*,), genetic epilepsy syndromes (*KCNA2, SCN2A, PRRT2, TBC1D24*), GLUT-1, mitochondrial disorders (*PDHA1, PDHX, TPK1, DARS2, ACO2*), metabolic disorders [aminoacidopathies (Maple syrup urine disease: *BCKDHA, BCKDHB, DBT*; Hartnup disease: *SLC6A19*), urea cycle defects (type I citrullinemia: *ASS1*), thiamine metabolism defects (thiamine pyrophosphate deficiency: *TPK1*) biotin metabolism (biotinidase deficiency: *BTD*),] and others (*KCND3, NALCN*, FHM2/*ATP1A2, PACS1, CEP290*). Some of these might explain prior classical familial EA cases that were negative for EA1 and EA2 and genetic loci of other EAs [1,6,87]. Three of them (SCA-27/*FGF14*, SCA-42/*CACNA1G*, and *SCN2A*) have been proposed to be categorized as EA9 or EA10.

#### SCA-14 (*PRKCG*)

SCA-14 is a dominantly inherited slowly progressive ataxia, sometimes accompanied by parkinsonism, dystonia, myoclonus and cognitive impairment. It is caused by mutations in *PRKCG* gene encoding protein kinase C gamma (PRKγ). *PRKCG* mutations may also present with adult-onset episodic ataxia, with a frequency of 1/14 *PRKCG*-positive patients [88].

#### SCA-27 (*FGF14*)

SCA-27 is a late-onset progressive ataxia with parkinsonism, postural tremor and titubation; 20% have coexistent episodic ataxia [89]. It is caused by mutations in *FGF14* gene which encodes Fibroblast Growth Factor 14. This protein is highly expressed in the brain, especially Purkinje cells, where it interacts with voltage-gated Na+ channels to regulate neuronal excitability [89]. Isolated EA is also reported to be caused by heterozygous FGF14 gene mutations [75,89,90,91,92]. Onset age ranges widely from early childhood to adulthood. Attacks may be accompanied by vertigo, dizziness, unsteadiness, with interictal nystagmus and tremor. Attacks are highly variable, lasting seconds up to several days. There are a variety of triggers; a fever trigger with a prolonged attack in a young child can mimic febrile cerebellitis. Attacks may respond to acetazolamide, and may improve with age. Developmental delay and paroxysmal dyskinesia have also been observed [90]. Some authors suggested designating this EA9.

#### SCA-42/epilepsy (*CACNA1G*)

*CACNA1G* encodes the pore-forming α1G subunit of T-type voltage gated calcium channel (VGCC). Mutations in *CACNA1G* cause generalized absence epilepsy and SCA42. A single family is reported with episodic vestibulocerebellar ataxia associated with a mutation in the *CACNA1G* gene [93]. There were attacks of dizziness, unsteadiness, headache and facial numbness, and head-movement induced vertigo. Attacks lasted up to several months in duration. Interictal examination showed cerebellar findings and bilateral vestibulopathy. The attack duration and absence of myokymia or tinnitus distinguishes this from EA3. Attacks were worsened by acetazolamide, and suppressed by carbamazepine. The authors proposed this be designated EA10.

#### AOA2 (*SETX*)

Mutations in *SETX* (senataxin) account for two separate clinical syndromes. Oculomotor apraxia type 2 (AOA2), an autosomal recessive spinocerebellar ataxia, with adolescent or early adult onset progressive ataxia, oculomotor apraxia, neuropathy, cerebellar atrophy, and elevated alpha-fetoprotein levels. Autosomal dominant juvenile-onset motor neuron disease (ALS4) is also characterized. There is a single case report of a 4 year old boy presenting with isolated severe EA attacks lasting 20–30 minutes, and intermittent mild impaired tandem gait between attacks [94]. Genetic testing excluded EAs 1,2,5,6. Whole exome sequencing (WES) identified a heterozygous deletion in *SETX* gene, possibly explaining the milder phenotype compared to homozygous mutations in AOA2.

#### CAPOS/RODP/AHC (*ATP1A3*)

Mutations in the *ATP1A3* gene mutation cause a broad spectrum of neurologic disorders. These include the clinical syndrome of cerebellar ataxia, areflexia, pes cavus, optic atrophy and sensorineural hearing loss (CAPOS), rapid-onset dystonia parkinsonism (RODP), and alternating hemiplegia of childhood (AHM) [95]. It may also present with paroxysmal ataxia triggered by fever, with attacks responsive to acetazolamide [96]. Dystonia, hypotonia, neuropsychiatric symptoms, cognitive impairment, and microcephaly may also be observed [97].

#### GLUT-1 (*SLC2A1*)

The *SLC2A1* gene encodes glucose transporter protein type 1 (GLUT1) which facilitates glucose transport across the blood-brain barrier, and is critical for brain energy. The GLUT-1 deficiency syndrome is a result of inadequate brain glucose transport. The main phenotype is a severe chronic neurologic disorder (microcephaly, developmental delay, early infantile seizures, ataxia) [98]. About 10% do not have this phenotype, and instead have milder paroxysmal variants often provoked by fasting or exercise, such as EA or paroxysmal exercise-induced dyskinesia (PED) [99]. Amongst 25 SLC2A1 carriers, 1 had EA [100]. The GLUT-1 spectrum disorder has grown to encompass other paroxysmal dyskinesias (PKD, PNKD), myotonia, migraine, hemiplegic migraine and episodic eye movements [100,101]. EA may be pure or with additional neurological findings [99,102,103]. A diagnostic test to assess for GLUT1 deficiency is spinal tap showing a low CSF to serum glucose ratio (hypoglycorrhachia). Treatment for GLUT1 deficiency is avoidance of triggers, or ketogenic diet to provide an alternative energy substrate for brain energy metabolism [98,104,105]. However EA attacks may also respond to acetazolamide, which may lead to misdiagnosis as EA2 [99,102].

#### Epilepsy spectrum disorder (*KCNA2*)

A spectrum of neurological disorders may be caused by mutations in the *KCNA2* gene, which encodes voltage-gated potassium channel Kv1.2. Early onset developmental and epileptic encephalopathy, intellectual disability, and ataxia are recognized. A milder phenotype of episodic ataxia, epilepsy, and complicated hereditary spastic paraplegia is reported [106,107].

#### Epilepsy spectrum disorder (*SCN2A*)

Mutations in *SCN2A* are associated with a spectrum of neurological disorders from benign to severe epilepsies, autism spectrum disorder and intellectual disability [108,109]. *SCN2A* encodes the alpha subunit of voltage gated neuronal Nav1.2 channel. Loss-of-function mutations result in severe epilepsy, intellectual disability and autism, whereas gain-of-function mutations cause benign familial neonate infantile seizures (BFNIS) with or without EA [110]. Patients with BFNIS have seizures before age 3 months which resolve in early life, and may later develop EA between age 10 months to age 14 years [1,108,111]. Cognitive outcome is mostly favorable in these cases [108]. Amongst cases with a more severe intellectual disability phenotype, EA appears to be uncommon [112]. Phenotypes may vary in family members, e.g. EA in infant and episodic hemiplegia in parent [113]. EA may be triggered by vaccinations, minor head trauma, and sleep deprivation [114,115]. Some but not all have a favorable response to acetazolamide [108]. Seizures, but not EA attacks, respond to Na-channel blockers (phenytoin, carbamazepine) suggesting different pathophysiologic mechanisms. Some authors have designated this EA9 [116].

#### PKD/epilepsy (*PRRT2*)

*PRRT2* (proline-rich transmembrane protein 2) mutations are responsible for a spectrum of paroxysmal neurological disorders. The 3 main phenotypes are paroxysmal kinesogenic dyskinesia (PKD), benign familial infantile convulsions (BFIC) and infantile convulsions and choreoathosis (ICCA) [117,118,119]. Other phenotypes include migraine, FHM and epilepsy [100]. EA appears to be a rare manifestation of *PPRT2* mutations. In 1 large study of 374 PRRT2-positive patients, episodic ataxia was only occasionally reported [120]. In another study of 182 EA patients, only one case was attributed to a *PRRT2* gene mutation [121]. MRI brain imaging performed in a *PRRT2* patient during an EA attack showed cerebellar diffusion restriction [122]. Most *PRRT2* mutations respond exquisitely to carbamazepine [123] so EA attacks due to *PRRT2* may cause diagnostic confusion with EA1. The *PRRT2* protein interacts with SNAP-25 and may play a role in synaptic transmission. Most mutations are loss-of-function, which may result in disrupted synaptic transmission and neuronal hyperexcitability. This does not explain phenotype variation, and no specific phenotype-genotype correlation is identified [124]. A handful of cases are reported with homozygous *PRRT2* mutations and a severe phenotype with episodic ataxia, intellectual disability, and infantile seizures [125,126,127].

#### Epilepsy spectrum/DOORS (*TBC1D24*)

Mutations in the *TBC1D24* presynaptic protein are associated with a neurological spectrum of epilepsy, chronic encephalopathy, DOORS (deafness, onychodystrophy, osteodystrophy, mental retardation and seizures), hearing loss, and myoclonus. Biallelic mutations of *TBC1D24* were found in an infant with EA and myoclonus, with a later finding of cerebellar atrophy in adolescence [128].

#### Mitochondrial disorders

EA cases are reported in mitochondrial disorders, such as pyruvate dehydrogenase complex deficiency, (*PDHx, PDHA1), TPK1, DARS2, MTATP6, ACO2* genes [4]. EA can be isolated or occur with other neurological abnormalities. Diagnostic clues are the presence of serum and CSF lactic acidosis. A mild presentation of fever-triggered EA attacks lasting 2 to 7 days, and a normal interictal exam, was observed in a young child with PDH deficiency [129]. Tests showed elevated serum and CSF lactate, and MRI brain showed dentate nucleus hyperintensity. Attacks responded to thiamine, levocarnitine, and alpha-lipoic acid. In comparison, homozygous or compound heterozygous mutations in *ACO2* (encodes mitochondrial aconitase 2 that catalyzes citrate to isocitrate) can cause a spectrum of disorders with often severe neurologic impairment. A recent report of 2 siblings with *ACO2* mutations had EA plus mild developmental delay and neuropsychiatric symptoms [130].

#### Unknown genes

A “late-onset EA” was reported in 2009 of 4 cases in a single 2-generation family but the gene is not known [87]. Onset was in the fifth or sixth decade. Phenotype severity was variable, with more severe cases exhibiting daily attacks with slowly progressive ataxia and poor acetazolamide response. Screening excluded *KCNA1* (EA1), *CACNA1A*, (EA2), and locus for EA2, EA5, EA6 and EA7.

### Secondary (acquired) episodic ataxia

There is a broad differential diagnosis for acute-onset recurring ataxia [131]. (Table 2). Secondary or acquired EA may resemble primary EA with regards to onset-age, attack variability, and interictal cerebellar findings, but are more likely to have abnormal laboratory and MRI imaging [132]. Many secondary causes are treatable, and collectively more common that primary EAs, so they are important to consider.

**Table 2:** Differential diagnosis for episodic ataxia.


Vestibular migraine

Migraine with brainstem aura

Peripheral vestibular disorders (e.g. BPPV, Vestibular neuritis, labyrinthitis, Meniere’s disease, acoustic neuroma, perilymphatic fistula)

Epileptic pseudoataxia

Toxicity (e.g. antiseizure medications, lead, alcohol)

Inflammatory (multiple sclerosis, postinfectious cerebellitis, Bickerstaff brainstem encephalitis, Miller-Fisher syndrome)

Paraneoplastic/autoimmune ataxia (CASPR2, NMDA-R, ANNA-1)

Vascular (TIA, ischemic stroke, hemorrhage, antiphospholipid syndrome, Kawasaki disease, neuro Behcet’s disease with brainstem and red nuclei involvement)

Tumor (posterior fossa or cerebellum, occult neuroblastoma)

Metabolic (e.g. maple syrup urine disease, pyruvate dehydrogenase deficiency, ornithine transcarbamylase deficiency, biotinidase deficiency, Hartnup disease, argininosuccinic aciduria)

Hypothyroidism

Paroxysmal dyskinesia

Functional neurologic disorder


The most common secondary disorders are transient ischemic attacks or stroke, multiple sclerosis or other immune-mediated disorders [133]. “Paroxysmal dysarthria and ataxia” (PDA) is a well-recognized phenomenon in multiple sclerosis, with stereotyped multiple daily episodes of sudden ataxia lasting seconds to minutes, attributed to ephaptic transmission [134]. This PDA syndrome can mimic genetic EA, and is also reported in immune-mediated diseases such as antiphospholipid syndrome, Bickerstaff’s/Bickerstaff-like encephalitis, certain autoimmune ataxias, and ischemic stroke [133,135,136]. This has been attributed to a lesion in the midbrain, near or in the red nucleus [134]. Other inflammatory disorders (postinfectious cerebellitis, Miller-Fisher syndrome) and vascular disorders (Behcet’s disease with brainstem and red nuclei involvement, Kawasaki disease) can present with prolonged attacks of acute ataxia. Structural lesions in the posterior fossa or cerebellum such as a tumor or occult neuroblastoma can present with recurrent ataxia.

Epileptic pseudoataxia may transiently occur after a seizure. Hypothyroidism can present with recurrent ataxic episodes, and responds to thyroxine. Toxins (e.g. alcohol, antiseizure medications, lead) can present with reversible acute ataxia. Metabolic disorders (e.g. maple syrup urine disease, pyruvate dehydrogenase deficiency, ornithine transcarbamylase deficiency, biotinidase deficiency, Hartnup disease, argininosuccinic aciduria, citrullinemia, thiamine pyrophosphate deficiency) causing EA usually present in childhood with severe neurologic symptoms, but may present in adults with much milder features [4]. Thiamine pyrophosphate deficiency has been reported in a small number of patients with EA, delayed development and dystonia, and may respond to thiamine supplementation [137]. Maple syrup urine disease may also have significant clinical or biochemical improvement with thiamine supplementation [137]. Citrullinemia is a rare recessive urea cycle disorder due to mutations in the *ASS1* (type I citrullinemia) gene which cause deficiency of arginosuccinate synthetase enzyme, necessary for catalyzing the formation of arginosuccinic acid from citrulline and aspartic acid. A typical presentation is a neonate with toxic hyperammonemia and progressive encephalopathy. Mild late-onset childhood or adult-onset forms with intermittent symptoms (ataxia, headache, stroke, intellectual disability, or encephalopathy) are reported. A case of citrullinemia presented in late childhood with brief EA attacks with fever, a normal interictal neurological exam, cerebellar atrophy, and elevated citrulline and ammonia blood levels [138].

Autoimmune ataxias are usually chronic, but three types to date may manifest with EA. CASPR2 (VGKC complex) can present with episodic ataxia and dysarthria, seizures and cognitive dysfunction. MRI brain may be normal or show medial temporal hyperintensity, with elevated CSF protein and positive CASPR2-IgG in serum and CSF [139,140,141]. It responds to immunotherapy. Anti-NMDA receptor autoimmunity can present with paroxysmal dysarthria-ataxia syndrome [142]. Anti-Hu (ANNA-1)-associated paraneoplastic limbic encephalitis presented in a child as episodic ataxia and progressive behavioral changes evolving to intractable epilepsy [143]. Iatrogenic intermittent ataxia may be provoked during deep brain stimulation programming [144]. Finally, functional ataxia may be suggested by incongruent examination findings, distractibility, and the presence of other functional signs.

### Differential diagnosis for episodic ataxia

EA can be mimicked by other paroxysmal disorders with stereotyped attacks of central or peripheral origin (e.g. vestibular migraine, migraine with brainstem aura, seizures, paroxysmal dyskinesias or benign paroxysmal positional vertigo). Patients with EA have been misdiagnosed with migraine, seizures, functional or anxiety disorders, resulting in premature diagnostic closure [22,55,57]. A personal or family history of epilepsy or migraine may have suggested these alternative more common diagnoses, rather than EA. It may also be difficult to distinguish chronic ataxia with stepwise exacerbations or stepwise decline (e.g. SCA 6), from EA with persistent cerebellar dysfunction. Suspicion for EA should be heightened with acetazolamide-responsive attacks.

### Approach to episodic movement disorders in the clinic: (See Table 3)

#### Clinical assessment

EA can be readily misdiagnosed or overlooked. In order to recognize, it is important to routinely include it in the differential diagnosis of spells, whether these are movement or non-movement based. A detailed history should include: onset age, triggers, duration, frequency, aura, baseline between spells, and response to treatment trials. Patient descriptors may pose challenges (e.g. episodic stiffening due to EA1 versus PKD attack, or EA with episodic cognitive impairment due to EA2 versus seizure). Events during early childhood development (e.g. infantile paroxysmal torticollis, episodic oculomotor dysfunction, BFNIS) may provide diagnostic clues for EA2. The interictal examination can offer clues to primary EAs when findings are present (e.g. nystagmus EA2, myokymia EA1). Ictal examination or a video of the attack can help reconstruct the phenomenology. A 3-generation family history is important to look for other paroxysmal neurologic disorders because of considerable phenotypic variability in families. Family history may appear negative with de novo mutations, false paternity, early death, or estrangement from biological family, deceptively pointing away from a genetic cause. Secondary EAs or mimicking conditions can be suggested by history, examination, and imaging findings. While functional features may suggest a non-genetic EA, functional embellishment of primary EA may lead to diagnostic uncertainty regarding the predominant etiology [22]. Diagnostic delay or misdiagnosis include patient factors (young children cannot give a history, atypical features) or clinician factors (bias towards alternative diagnosis, atypical or evolving phenotype). Diagnostic challenges may also arise where attack duration or triggers overlap between categories. For example, EA1 and EA2 attacks may both last hours and have permanent cerebellar signs, so one needs to look for interictal myokymia or nystagmus or ancillary tests to distinguish them.

**Table 3:** Approach to diagnosing EA disorders in the clinic.


History of episodic spells	Onset age, triggers, duration, frequency Other attack symptoms? e.g. vertigo, tinnitus, confusion Medical history of other paroxysmal disorders? Migraine, seizures 3-generation family history Baseline between spells Response to treatment

Neurological examination	At baseline, during a spell, video of spell

Electroencephalogram	Routine EEG with provoked spell, video EEG

MRI brain with and without contrast	Normal, cerebellar atrophy, other brain lesions

Laboratory tests (secondary etiology suspected)	Thyroid function, thiamine, anti-seizure medication levels, ammonia, citrulline, lead, ETOH, anti-GQ1B antibodies, sedimentation rate, C-reactive protein, lupus anticoagulant, anticardiolipin antibodies IgG/IgM, anti-beta-2 glycoprotein antibodies IgG IgM CSF cell count, glucose, protein, oligoclonal bands, MS profile Paired serum/CSF: glucose, lactate, CASPR2, anti-NMDA-R, ANNA-1

Genetic testing	Single-gene if classical phenotype and family history (e.g. EA1, EA2) Episodic ataxia panels Next-generation sequencing Whole exome sequencing


#### Investigations

Brain imaging looking carefully for cerebellar atrophy or other structural brain lesions can provide diagnostic clues for primary or secondary EA. Electroencephalography during a triggered attack can help differentiate EA from an epileptic event. However, baseline EEG abnormalities may be encountered in both EA2 and genetic epilepsies with EA. Laboratory testing (e.g. serum and CSF lactate or glucose can assist diagnosis of mitochondrial disorders or GLUT-1 syndromes respectively). For secondary cases, blood tests for metabolic disorders and toxins may be necessary.

#### Genetic testing

If one suspects a primary EA that is fairly classical for either EA1 or EA2, proceeding to single gene testing for *KCNA1* or *CACANA1A* may be an appropriate choice. In uncertain cases, a multigene EA panel could be used [145]. For atypical or complex cases, where other investigations have failed, next generation sequencing or whole exome sequencing has diagnostic utility to detect a causal gene mutation [21]. Reaching a specific genetic diagnosis can provide clinical value: guide treatment, reduce unnecessary investigative tests, aid genetic counselling, and further refine phenotype-genotype profiles [21,52,109].

#### Treatment

For cases that resemble EA1 or EA2, one may proceed directly to first-line treatment (antiseizure medication or acetazolamide, respectively), without requiring genetic confirmation [145]. If unsuccessful, consider second-line treatment trials. Ultimately, genetic testing is gold-standard for the diagnosis to guide appropriate treatment and long-term management. Many of the primary EAs, other genetic causes of EA, and secondary forms of EA are treatable. (See Table 4).

**Table 4:** Treatable causes of episodic ataxia.


	GENE OR MECHANISM	TREATMENT

Primary EA		

EA1	*KCNA1*	Carbamazepine, other anticonvulsant drugs, (Acetazolamide)#

EA2	*CACNA1A*	Acetazolamide, 4-AP, dalfampridine, fampridine

EA3	unknown	Acetazolamide

EA4	unknown	Gabapentin

EA5	*CACNB4*	Acetazolamide

EA6	*SLC1A3*	Acetazolamide

EA8	*UBR4*	Clonazepam

Other genetic EA		Carbamazepine

EA9?	*FGF14*	Acetazolamide

EA10?	*CACNA1G*	Carbamazepine

EA9?	*SCN2A*	(Acetazolamide)#

	*PRRT2*	Carbamazepine

	*SLC2A1*/GLUT-1 deficiency	Ketogenic diet, Carbamazepine

	*ATP1A3*	Acetazolamide

Secondary EA		

Metabolic	Hypothyroidism	Thyroxine

	Thiamine pyrophosphate deficiency	Thiamine

	Thiamine transporter (*SLC19A3*)	Biotin, Thiamine

	Biotinidase deficiency (*BTD*)	Biotin

	Hartnup disease	Niacin supplement

	Maple syrup urine disease (*BCKDHA, BCKDHB, DBT*)	Dietary restriction of branched-chain amino acids, Thiamine supplement

	Ornithine transcarbamylase deficiency	Dietary restriction of nitrogen intake

	Pyruvate dehydrogenase deficiency (*PDHX, PDHA1*)	Thiamine, alpha-lipoic acid, ketogenic diet, dichloroacetate

	Mitochondrial	Mitochondrial cocktail

Inflammatory	Multiple sclerosis	Steroids, disease-modifying therapies

	Behcet’s	Immunomodulatory treatment

	Kawasaki disease	High-dose aspirin, IVIG, steroids

Autoimmune	Autoimmune (CASPR2, anti-NMDA-R, anti-Hu/ANNA-1)	Steroids, immunomodulatory treatment

	Bickerstaff brainstem encephalitis	IVIG, Plasmapheresis

Miller-Fisher syndrome	IVIG, Plasmapheresis

Toxic	Toxicity (lead, alcohol, AEDs)	Discontinue medications/toxic exposure

Vascular	TIA/Stroke	Secondary stroke prevention, rehabilitation

Epilepsy	Epileptic pseudoataxia	Antiseizure medications

Iatrogenic	Thalamic deep brain stimulation	Programming adjustment

Functional	Functional	Functional motor and cognitive rehabilitation


# A response is not reliably observed.

## Discussion

EA encompasses a complicated group of disorders, with continually expanding phenotypes and a growing list of causes. Over the past decade, genetic advances appear to have increased, rather than simplified, diagnostic difficulty. There has been further expansion of EA1 and EA2 phenotypes, identification of about 20 other genetic causes of EA, and reporting of new secondary causes of EA. Moreover, for EAs 3, 4, 5, and 7 many questions remain unanswered, as no new families have been identified or the gene remains unknown. Autopsy findings in EA4 open up the possibility that this could have been caused another familial ataxia, such as SCA6. Recent proposals to assign EA9 (*FGF14, SCN2A*) and EA10 (*CACNA1G*) are yet to be formally accepted.

The genes underpinning EA can exhibit significant overlap with other neurological paroxysmal phenomena, such as epilepsy and migraine. For example, EA and epilepsy associations include EA1 (*KCNA1*), EA2 (*CACNA1A*), EA5 (*CACNB4*), EA6 (*SLC1A3*), *SCN2A, KCNA2, ATP1A3, SLC2A1*, and *PRRT2* [116]. This suggests a shared pathophysiological basis, and advances into the underpinnings of EA may translate into better understanding for these other paroxysmal disorders.

The phenotype is often not accurately predicted by the underlying genotype. Within the same family there can be large variability in attack frequency, disease severity, and treatment response, despite the same genotype. It is presumed that the phenotype must therefore be modulated by environmental factors, modifier genes, or age-dependent expression [146]. This seems plausible as these are episodic (not fixed) disorders, and environmental modifiers are already illustrated by the presence of attack triggers. A study suggested *UBR4* and *SLC1A3* may act as genetic modifiers with a synergistic effect on *CACNA1A* mutation [74]. Plausibly, these modifier genes could be developed as a therapeutic target or a new precision therapy [116]. For example, several genes have identified in EA1 mice that modify the epilepsy phenotype in EA1 mice, so potentially this could be adapted for EA treatment [3]. Moreover, the age-dependent expression observed with some of these disorders, such as *CACNA1A*, may simply reflect properties of neurologic channelopathies, where different phenotypes can arise at different ages, and the adult phenotype may differ considerably from the childhood syndrome [147]. It is also transpiring that the infantile *CACNA1A* paroxysmal phenotypes are not so benign as their names would suggest, given their increasing association with chronic neuropsychiatric impairment.

Improved understanding of genotype-phenotype relations using molecular and electrophysiological study in animal models and patients may result in better precision medicine. A machine-learning method was recently applied to 47 patients with 33 unique variants in *CACNA1A* (pathogenic or likely pathogenic) to predict LOF or GOF mutations [148]. The severity score was significantly higher for GOF variants, S5/S6 helices variants and pVal1392 Met variant. This was interpreted as demonstrating broad disease severity in *CACNA1A* disease and that clinical phenotypes likely reflect diverse molecular phenotypes [148]. A recent study used gene interaction networks to investigate common gene signatures associated with paroxysmal phenotypes of ataxia, migraine, epilepsy and other movement disorders [149]. Nineteen candidate genes were used to create an interaction network, which further revealed 39 associated genes (including *KCNA1, SCN2A, CACNA1A*, and *CACNB4*). The meta-regression analysis showed the strongest association of *SCN2A* with genes in neurodevelopmental disorders, and *KCNMA1* as a common gene signature with a link to epilepsy, movement disorders and wide paroxysmal neurologic presentations. Identifying gene interactions may help future drug targets [149].

Using advanced genetic testing may be the crucial step for undiagnosed hereditary EAs, although this can create its own challenges. Genetic testing may be restricted by methodology or techniques, such that the pathogenic gene was omitted, or mutations may not be adequately detected (e.g. repeat expansion, microdeletion). If mutations are detected, there may be additional challenges because of broad phenotype variability, poor genotype-phenotype correlations, or a large number of VUS identified (e.g. *CACNA1A*) [120]. Frequently, no functional study of a mutated protein is performed so we cannot be certain of its pathogenicity [1]. This could be improved by accessible functional read-outs, particularly for atypical cases or with cheaper or more readily available next-generation testing [40]. On a global scale, there may be underdiagnosis of genetic EAs in resource-poor settings. Apart from EA1 and EA2, most other EA reports reflect Caucasian cases. A recent Korean study found genetic heterogeneity in 33/39 EA patients, when examining a range of suspected pathogenic mutations in *CACNA1A, SLC1A3, UBR4, SCNA1, TTBK2, TGM6, FGF14* and *KCND3* [74]. However more studies are needed to update global genetic differences of EA, similar to the SCAs and genetic parkinsonisms.

Prior to advances in genetic testing, all EAs were thought to be channelopathies. Genetic mutations in *KCNA1, CACNA1A, CACNB4, SLC1A3, SCN8A, KCNMA1*, and *ATP1A3* genes that encode ion channels lend support to the channelopathy theory [150]. Moreover, the overlap of movement disorders, migraine and epilepsy is often described in channelopathy disorders [151]. However other EA genes do not encode ion channels, suggesting alternative mechanisms [152]. There is evidence to suggest that the presynaptic terminal is involved, as *PRRT2* and *SLC1A3* likely act on the presynaptic terminal, and both *KCNA1* and *CACNA1A* are presynaptic [100]. *KCNA1* and *CACNA1A* have the highest levels of expression in the cerebellum, and in frontal, temporal and occipital cortices, compared with GLUT-1/*SLC2A1*, so the regional effect of vesicle release could explain phenotypic differences. This may be why *KCNA1* and *CACNA1A* are more likely to present with ataxia than GLUT-1. This regional effect may not explain their other phenotypes such as migraine. Instead, they might be attributed to the consequences of dysregulated presynaptic terminals. Mice models of migraine with single gene mutations have shown increased glutamatergic neurotransmission and cerebral hyperexcitability, which may reflect abnormal neurotransmitter release from the presynaptic terminal [100,153].

The current classification system and diagnostic algorithm for EA frequently designates EA1, EA2 and others. This seems too simplistic given the current number of genes identified. Moreover, clinical prediction for the underlying gene is unreliable, as even classical EA1 and EA2 phenotypes can be *KCNA-1* and *CACNA1A* negative respectively. Empiric treatment may also result in misdiagnosis, e.g. acetazolamide responsive GLUT-1. It is likely time to reconsider the nosology for EA.

The simplest solution might be to ascribe EA numbers to all the genes identified to date for EA. This would be similar to the SCAs, which currently number 50. The caveat is that some genes are more commonly associated with non-EA syndromes. Another suggestion is to classify EA by its mutation. This has been proposed for the PKDs e.g. PKD-*PRRT2* or PKD-*SCN8A* [150]. This could be readily used for EA, e.g. EA-*PRRT2*, EA- *SLC2A1*, EA-*SLC1A3*, etc. However, a limitation is that this could only be used if a causative gene is identified. Instead, we could consider diagnostic algorithms proposed for the paroxysmal dyskinesias, another episodic disorder. One suggestion uses a 2-axes system. Axis 1 is clinical classification by trigger to establish PKD, PNKD, or PED, and Axis 2 classification is the presence or absence of 4 causative genes (PRRT2, MR-1, KCNMA-1, and SLC2A1); if negative, further testing can be pursued [152]. However genetic EAs share multiple triggers, may not be reliably clinically distinguished by a single clinical feature alone, and have a wider genetic spectrum.

Therefore, turning to the dystonia classification system may provide a better model for EA classification. This also combines 2 axes: clinical characteristics and etiology, with the goal of helping guide diagnosis and treatment [154]. Adapting this model for EA, Axis 1 clinical characteristics could include age at onset, attack duration, simple (dysarthria-ataxia) or complex attack, interictal exam (normal or abnormal), and other neurologic comorbidities (e.g. epilepsy, intellectual impairment) and Axis 2 etiology could include nervous system pathology, and whether inherited, acquired or unknown. (See Tables 5 and 6). An additional category to consider in Axis 1 etiology is empiric treatment response e.g. acetazolamide- responsiveness in many EAs, *FGF14*, and *ATP1A3* but absent in others. These combined clinical aspects may suggest EA syndromes to help guide genetic diagnosis and treatment. For example, childhood-onset short duration simple EA attacks, with a normal interictal exam and normal imaging could suggest *KCNA-1* or *PRRT2*, whereas the presence of abnormal imaging could suggest *PDHx*, or interictal ataxia could suggest *SETX*. Adult-onset long duration EA attacks may suggest *CACNA1A, SLC1A3*, or *FGF14*. Adult-onset short duration simple EA attacks, with abnormal interictal exam and imaging could be MS or autoimmune ataxia. Future research into analysis of this proposal would be of interest to assess if it could improve clinical diagnosis and genetic prediction.

**Table 5:** Proposed Axis 1 clinical characteristics for EA.


CLINICAL CHARACTERISTICS OF EA (AXIS 1)				OTHER NEUROLOGIC DISORDERS

AGE AT ONSET	ATTACK DURATION	SIMPLE OR COMPLEX ATTACK	INTERICTAL FEATURES	

Infancy (birth to age 2) Childhood (3–12 years) Adolescence (13–20 years) Early adult (21–40 years) Late adult (>40 years)	Short (seconds-minutes) Moderate (hours) Long (days or longer)	Paroxysmal ataxia-dysarthria – isolated OR – with other features tinnitus – vertigo – nystagmus – brainstem – neuromuscular – migraine – other	Nystagmus Myokymia Other cerebellar signs Tremor Other neurologic signs	Migraine Epilepsy Intellectual impairment Deafness Autism Paroxysmal disorder of infancy


There are five proposed clinical descriptors in Axis 1 (age at onset, attack duration, attack complexity, interictal features, and other neurologic comorbidities).

**Table 6:** Proposed Axis 2 etiology of EA.


ETIOLOGY OF EA (AXIS 2)			

NERVOUS SYSTEM PATHOLOGY	INHERITED OR ACQUIRED		

	INHERITED	ACQUIRED	UNKNOWN

None Evidence of degeneration (Cerebellar atrophy) Evidence of structural lesions	Autosomal dominant Autosomal recessive Mitochondrial	Inflammation Demyelination Vascular Drugs Toxic Metabolic Neoplastic Paraneoplastic Functional	Sporadic Familial


The etiology axis is subdivided into neuroimaging findings, and the identification of a genetic or acquired cause underlying episodic ataxia. For example, an etiological description of an EA case could be ‘evidence of degeneration’ and an ‘autosomal dominant pattern, for EA2 (EA-*CACNA1A*), or ‘evidence of structural lesion’ and acquired cause for multiple sclerosis.

A final alternative strategy, also borrowing from PKDs, is to consider grouping EAs into categories by presumed pathogenic mechanism: i.e. channelopathies, neurotransmission synaptopathies, brain energy transportopathies, to create a new classification system [150].

Most EAs are treatable or even curable, so it is important to correctly diagnose them. There are now four effective treatments for EA2 include long-acting formulations of 4-aminopyridine (dalfampridine and fampridine) in addition to acetazolamide and 4-AP. Novel observations of sleep-alleviated EA2 attacks may suggest innovative treatment modulators. Many genetic EAs and GLUT-1 respond to acetazolamide. A trial of thiamine supplementation could be considered in cases suspicious for disorders of thiamine metabolism and PDH complex disorders.

## Conclusions

Episodic ataxias may be overlooked or misdiagnosed for a variety of reasons, including phenotype-genotype variability, clinical overlap with primary and secondary causes, and common mimicking disorders. As many primary EAs and secondary EAs are highly treatable, it behooves us to make the correct diagnosis, and to consider EA in the differential diagnosis of paroxysmal neurologic symptoms. EA1 and EA2 phenotypes have greatly expanded, and there are now several treatment options for EA2. There are unanswered questions regarding the entities of EA3, EA4, EA5 and EA7 despite the increased availability of genetic testing, in contrast with additional cases found carrying EA6 and EA8 genes. For classical phenotypes of EA1 and EA2, empiric treatment and single gene pathways can be used. For atypical phenotypes, more comprehensive evaluations and next generation genetic testing may be required for diagnosis. The proposed updated EA classification system and diagnostic algorithm may help better classify the growing list of genetic and secondary causes of EA to date.
