# Correction: Episodic Ataxias: Primary and Secondary Etiologies, Treatment, and Classification Approaches

**DOI:** 10.5334/tohm.1089

**Published:** 2025-08-25

**Authors:** Anhar Hassan

**Affiliations:** 1 Beacon Hospital, Sandyford, Dublin 18, Ireland; 2 UCD School of Medicine, University College Dublin, Belfield Dublin 4, Ireland; 3 Beaumont Hospital, Beaumont, Dublin 9, Ireland

**Keywords:** ataxia, cerebellum, episodic, paroxysmal, genetic, primary, secondary, treatment

## Abstract

This article details a correction to: Hassan A. Episodic Ataxias: Primary and Secondary Etiologies, Treatment, and Classification Approaches. *Tremor and Other Hyperkinetic Movements*. 2023;13(1):9. DOI: https://doi.org/10.5334/tohm.747

## Correction

The article “Episodic Ataxias: Primary and Secondary Etiologies, Treatment, and Classification Approaches” by Hassan A. contains an error in table 4 [1].

In table 4, the treatment for SLC2A1/GLUT-1 deficiency is listed as “Ketogenic diet, Carbamazepine”.

This should state the treatment is: “Ketogenic diet, Acetazolamide”.

Listing Acetazolamide is consistent with the rest of the discussion and references given in the article.
